# A new stepwise approach to minimize phrenic nerve injury during cryoballoon pulmonary vein isolation

**DOI:** 10.1007/s10840-024-01953-1

**Published:** 2024-12-20

**Authors:** K. Phkhaladze, H. Omran, T. Fink, V. Sciacca, D. Guckel, M. Khalaph, M. Braun, M. El Hamriti, J. Thale, G. Nölker, J. Vogt, C. Sohns, P. Sommer, G. Imnadze

**Affiliations:** 1 Clinic for Electrophysiology, Herz- und Diabeteszentrum NRW, Ruhr-Universität Bochum, Bad Oeynhausen, Germany; 2 Clinic for General and Interventional Cardiology/Angiology, Herz- und Diabeteszentrum NRW, Ruhr-Universität Bochum, Bad Oeynhausen, Germany; 3 Clinic for Internal Medicine and Cardiology, Klinikum Osnabrück, Osnabrück, Germany; 4 Department for Internal Medicine, Nephrology and Cardiology, Christliches Klinikum Unna, Unna, Germany

**Keywords:** Cryoballoon ablation, Atrial fibrillation, Phrenic nerve injury, Ablation, Pulmonary vein isolation

## Abstract

**Background:**

A phrenic nerve injury (PNI) during cryoballoon (CB) pulmonary vein isolation (PVI) continues to represent a limitation of this technique. The objective of this study was to develop a novel technique with the aim of reducing the incidence of PNI.

**Methods:**

We performed a retrospective analysis of data from two hospitals in patients with symptomatic, drug-resistant atrial fibrillation (AF) over 7 years to evaluate the incidence and clinical characteristics of PNI during cryoballoon PVI. Patients in the intervention group were treated with a new technique consisting of the following consecutive steps: (A) phrenic nerve stimulation near stimulation threshold instead of 10 V stimulation; (B) advanced ablation to the right superior pulmonary vein (PV) using a pre-freezing technique; (C) “pulling away” of the CB after vein isolation and/or after reaching − 40 °C for both right PVs. Two subtypes of PNI were studied: persistent (no recovery to discharge) and transient (recovery to discharge) PNI.

**Results:**

Nine hundred patients with a mean age of 62.3 (± 10.9) years (38% female) were analyzed. Transient PNI occurred in 8/250 patients (3.2%) in the intervention group compared to 39/750 patients (6%) in the control group (*p* = 0.09). Persistent PNI occurred in one patient (0.4%) in the intervention group compared to 18 (2.8%) in the control group (*p* = 0.03). Any PNI occurred in 9 patients in the intervention group (3.6%) compared to 57 patients (8.8%) in the control group (*p* = 0.008).

**Conclusion:**

In this retrospective analysis, a new cryo-PVI technique significantly reduces the incidence of PNI, particularly persistent PNI.

**Graphical Abstract:**

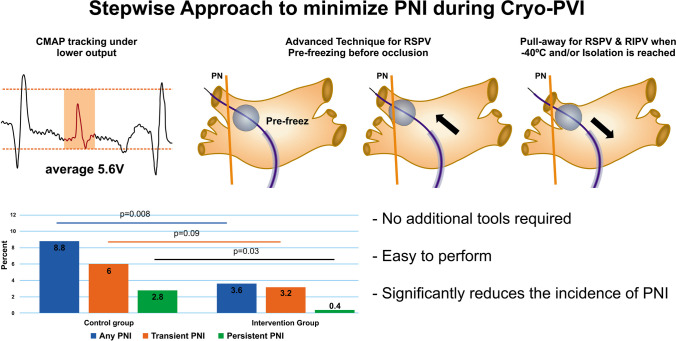

## Introduction

Atrial fibrillation (AF) is the most common type of cardiac arrhythmia, with a 1.5- to twofold increased risk of death. The prevalence of AF among the world population has increased over the past 20 years. The overall lifetime risk of this arrhythmia is about 15–40% [1]. Over the past decades, several catheter ablation technologies have been developed to significantly reduce the risk of arrhythmia recurrence. Radiofrequency (RF)-guided point-by-point pulmonary vein isolation (PVI) was the initial standard of care as first-line therapy for the symptomatic treatment of drug-refractory AF [2]. Following the development of new generation cryoballoon (CB) catheters, cryoballoon ablation has proven to be an equally effective therapeutic option for rhythm control in patients with AF, according to several recent studies. However, serious noncardiac events related to the procedure, such as phrenic nerve injury (PNI), were frequently identified. PNI is the most commonly observed complication of cryoballoon-guided PVI, with an incidence of 1.1–6.2% [3–5].

The main goal of our study was to develop a new concept that could significantly reduce the incidence of PNI during cryoballoon treatment of resistant AF. We have developed a new step-by-step technique for early detection and prevention of phrenic nerve damage.

## Methods

### Study Population

The study enrolled 900 consecutive patients who underwent cryoballoon pulmonary vein isolation for drug-resistant symptomatic AF at the Heart and Diabetes Center North Rhine-Westphalia, Germany, and the Klinikum Osnabrück, Osnabrück, Germany. This retrospective observational study followed patients who underwent an initial CB-PVI procedure between 2016 and 2023. Patients in the intervention group who underwent treatment with the new technique were enrolled from 2019. The study collected baseline, procedural, and follow-up data. The study was conducted in accordance with the principles outlined in the Declaration of Helsinki and approved by our local institutional ethics committee. The procedure in both groups was performed by three operators. Another two electrophysiologists had only performed procedures for the control group. All operators had experience of more than 100 procedures.

### Procedural management

Prior to the procedure, intracardiac thrombus was excluded by preprocedural transesophageal echocardiography (TEE). The majority of patients underwent computed tomography (CT) or cardiac magnetic resonance imaging to visualize detailed anatomy of the left atrium (LA) and pulmonary veins (PVs). Patients who were treated with Vitamin-K-antagonists maintained their daily anticoagulation adjusted to the procedure with a target International Normalized Ratio of between 2.0 and 3.0. In contrast, patients treated with novel oral anticoagulants (NOACs) discontinued medication uptake one half-life prior to the ablation procedure and resumed after a 4-h time interval. Exclusion criteria included any contraindications to the procedure, uncontrolled heart failure, and contraindications to anesthesia.

### Cryoballoon-based pulmonary vein isolation

The procedures were conducted under conscious sedation using propofol and analgesia with fentanyl as needed. Intravenous heparin was administered to maintain an activated clotting time (ACT) of > 300 s throughout the procedure.

Two right femoral vein punctures were performed. A 10-pole steerable diagnostic catheter (Inquiry™, Abbott, USA) was used for coronary sinus intubation, right ventricular rapid pacing, and phrenic nerve (PN) pacing. The procedure utilized a 28 mm 2nd generation CB (Arctic Front Advance, Medtronic, Minneapolis, MN, USA). After transseptal puncture, the balloon catheter was advanced into the LA through a steerable sheath (12-F FlexCath advance Medtronic, Minneapolis, MN, USA). PV mapping was performed using a multipolar mapping catheter (Achieve Advance Mapping Catheter, Medtronic, Minneapolis, MN, USA).

The transseptal puncture (TSP) was performed under fluoroscopy or intracardiac echocardiography (ICE) guidance in the control group until 2019. In the intervention group, TSP was guided solely by fluoroscopy.

After fluoroscopy-guided TSP, the control group underwent pulmonary vein angiography using the standard method or angiography under rapid pacing of the right ventricle. In all patients in the intervention group, angiography was performed under rapid pacing of the right ventricle, as previously reported [6]. Pulmonary vein occlusion was confirmed by PV angiography with the balloon inflated and placed at the PV ostia prior to each freezing cycle. The interventional group used the algorithm of time to isolation (TTI) plus 120 s, while the control group conducted a full freeze of 180 or 240 s. Permanent PVI (entry and exit block) was confirmed after a 20-min waiting period. Prior to ablating the septal PVs, a diagnostic catheter was inserted into the superior vena cava to stimulate the phrenic nerve. Compound motor action potentials (CMAP) from the right diaphragm were recorded along the abdominal palpation as described previously [7]. If the CMAP amplitude decreased by more than 30%, the cryoapplication was immediately stopped. To ensure proper phrenic nerve capture, we also observed diaphragmatic contraction under fluoroscopy.

### Definition of the phrenic nerve injury

We defined any PNI resulting in cessation of cryo application as an event, this included a decrease in CMAP. We defined PNI as transient if it recovered before the end of the procedure or before discharge. If PNI was sustained at discharge, we defined it as persistent.

### New Steps to Prevent PNI

In the intervention group, additional safety measures were taken during the ablation of the right pulmonary veins. Unlike the control group, the intervention group initially targeted the septal veins and the following steps were performed:

- **Step 1—Decrease PN stimulation output**
  The PN stimulation threshold was detected for all patients in the intervention group. Instead of continuous pacing with an amplitude of 10 V, we stimulated the phrenic nerve close to the pacing threshold with double output (Fig. 1).
- **Step 2—Pre-freezing for RSPV**
  **Fig. 1:**
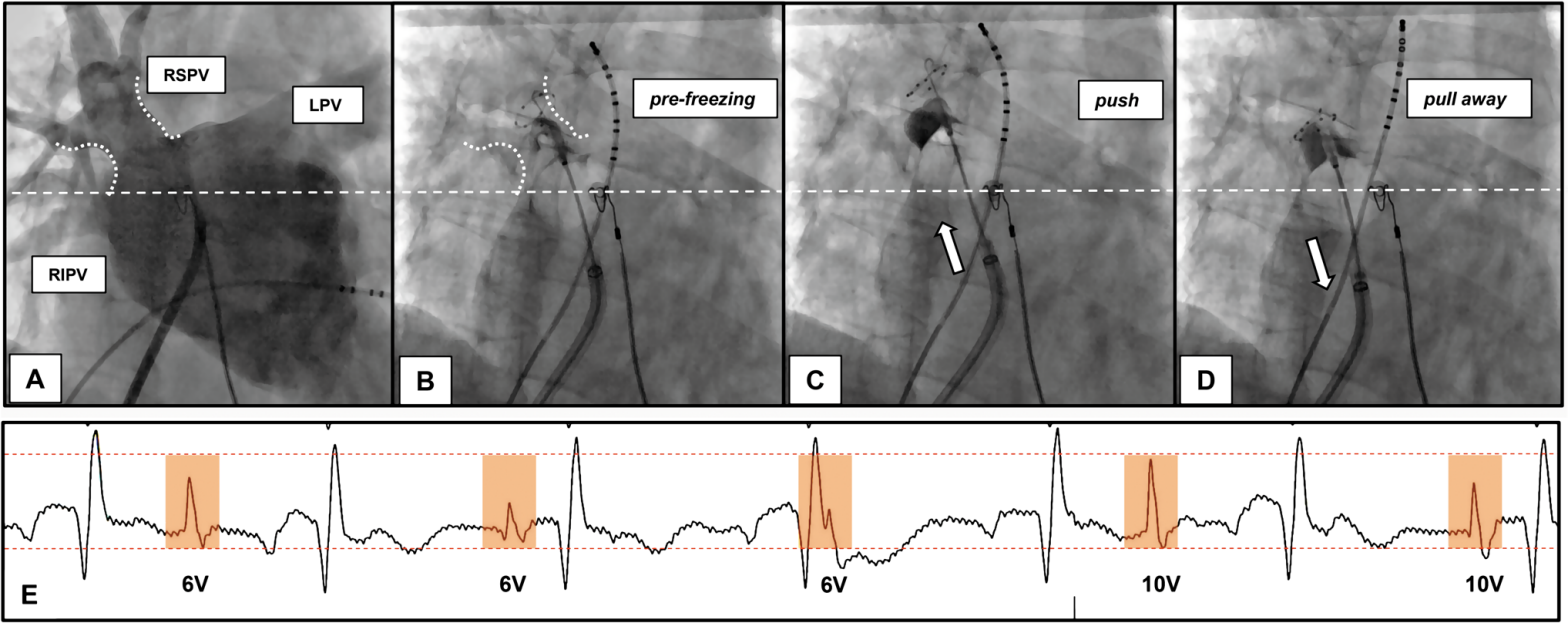
The consecutive steps of a new approach. All fluoroscopy pictures are presented in RAO 10° angulation, which is preferred over AP angulation because it provides better view of RIPV ostia. **A** Angiography of the PVs under rapid pacing from RV. Pre-freezing of the balloon close to the RSPV ostia (**B**) and pushing the balloon towards the ostia with simultaneous contrast injection until occlusion of the vein is verified (**C**). **D** After achieving −40 °C or isolating the vein, the pull-away maneuver (and hold until the end of the application) was performed. **E** The significance of PN stimulation with lower output, closer to the PN stimulation threshold, is demonstrated. The CMAP potential (colored blocks) decreases gradually when stimulated with 6 V/2.0 ms output (in this case, the PN stimulation threshold was 3 V/2.0 ms). Increasing the output to 10 V/2.0 ms immediately results in a higher CMAP potential, even higher than the initial potential under output with 6 V/2.0 ms. The application was immediately stopped despite high CMAP amplitude during stimulation with maximum output
  The pre-freezing technique was used to occlude and ablate the right superior pulmonary vein (RSPV). The approach involved inflating the cryoballoon in close proximity to the RSPV ostia, initiating the cryotherapy and then advancing the balloon towards the ostium under simultaneous angiography until occlusion was confirmed (Fig. 1).
- **Step 3—Pull away for septal PVs**
  Once the temperature reached − 40 °C and/or the PV was isolated, a “pull-away” maneuver was performed on both right-sided PVs under continuous freezing. This maneuver is similar to the pull-back maneuver, but the position is held until the end of the procedure (see Fig. 1).

### Postprocedural care and follow-up

After PNI was detected, each affected patient underwent a chest x-ray to determine the recovery status of the phrenic nerve before hospital discharge. Additionally, all patients underwent transthoracic echocardiography to exclude any possibility of fluid accumulation in the pericardium. Typically, patients were discharged the day after the procedure.

Anticoagulants were prescribed for a minimum of 3 months after the CB-PVI procedure. Lifelong anticoagulation was recommended according to the individual CHA2DS2-VASC scores. All patients were scheduled for follow-up visits after 3, 6, and 12 months.

### Statistical analysis

Statistical analyses were performed using IBM SPSS V29 (IBM Corporation, Armonk, NY, USA). Data are expressed as percentages for categorical variables and as mean ± standard deviation (SD) or median ± interquartile range (IQR) for continuous variables. We compared continuous variables using Student’s *t*-test and Mann–Whitney *U* test as appropriate. Comparisons of categorical variables between groups were performed by Pearson’s X^2^ test, for expected frequencies < 5 by two-sided Fisher’s exact test. Logistic regression analysis was implemented to adjust for other covariates. Univariate analyses were initially performed and all parameters with *p* < 0.1 were then included in multivariate analysis. All *p*-values were two-sided, and statistical significance was assumed at a *p*-value of 0.05.

## Results

Between 2016 and 2023, 900 patients suffering from drug-resistant AF underwent PVI using the cryoballoon technique. The control group consisted of 650 patients. In the intervention group, 250 procedures were performed using our novel stepwise approach. The study population had an average age of 62.3 ± 10.9 years and 37.7% of the patients were female. 52.1% had paroxysmal AF, while 47.9% had a history of persistent AF. The most common comorbidities in the study population were myocardial infarction (12.6%), followed by diabetes (11.6%), chronic kidney disease (3.9%), and chronic obstructive pulmonary disease (3.0%). There were no significant differences in baseline characteristics between the two groups, except for the higher age in the intervention group (Table 1). There was a significant difference in procedural data between the two groups. The intervention group had lower procedural time and contrast amount, while the control group had lower fluoroscopy time and radiation dose. Although there was no difference in the lowest temperature for all veins, the intervention group had a significantly lower time for cryo application compared to the control group (Table 2).

**Table 1:** Baseline characteristics of intervention and control groups

	Overall	Intervention group	Control group	*p*-value
*n*	900	250	650	
Age	62.3 ± 10.9	65.3 ± 10.3	61.9 ± 11.0	< 0.001
Female	342 (38%)	105 (42%)	237 (36.5%)	0.13
BMI	28.3 ± 4.9	28.3 ± 4.9	28.3 ± 4.8	0.99
History of MI	113 (12.6%)	37 (14.8%)	76 (11.7%)	0.21
Diabetes	104 (11.6%)	23 (9.2%)	81 (12.5%)	0.17
COPD	30 (3.0%)	12 (4.8%)	18 (2.8%)	0.13
CKD	35 (3.9%)	9 (3.6%)	26 (4%)	0.8
EHRA	2.4 ± 0.8	2.4 ± 0.7	2.3 ± 0.8	0.11
Paroxysmal AF	569 (52.1%)	127 (50.8%)	342 (52.6%)	0.63
PM	22 (2.4%)	7 (2.8%)	15 (2.3%)	0.67

*BMI* body mass index, *MI* myocardial ischemia, *COPD* chronic obstructive pulmonary disease, *CKD* chronic kidney disease, *EHRA* European Heart Rhythm Association classification, *AF* atrial fibrillation, *PM* pacemaker

**Table 2:** Periprocedural characteristics of intervention and control groups

	Overall	Intervention group	Control group	*p*-value
Procedural time (min)	79.8 ± 19.6	64.6 ± 20.8	88.5 ± 12.4	< 0.001
Fluoroscopy time (time)	8.7 ± 20.7	12.1 ± 7.9	7.4 ± 23.8	0.003
Radiation dose (Gy*cm^2^)	928 ± 1397	1158 ± 1668	840 ± 1267	0.007
Contrast volume (mL)	56.4 ± 19.1	44 ± 15.9	61 ± 18	< 0.001
RSPV				
Freeze duration	227.1 ± 61.2	191 ± 48	240 ± 61	< 0.001
Lowest temperature	− 48 ± 9.7	− 47.8 ± 9.6	− 48.0 ± 9.5	0.82
RIPV				
Freeze duration	250.5 ± 79.5	200 ± 47	270 ± 80	< 0.001
Lowest temperature	− 45.9 ± 10.1	− 46.5 ± 9	− 45.7 ± 10	0.3
LSPV				
Freeze duration	227.6 ± 49.5	199 ± 46	239 ± 47	< 0.001
Lowest temperature	− 46 ± 9.3	− 46.8 ± 9.9	− 45.7 ± 8.9	0.1
LIPV				
Freeze duration	237.7 ± 77	186 ± 49	257 ± 76	< 0.001
Lowest temperature	− 42.4 ± 11.1	− 43.9 ± 13.6	− 41.9 ± 9.7	0.01

*RSPV* right superior pulmonary vein, *RIPV* right inferior pulmonary vein, *LSPV* left superior pulmonary vein, *LIPV* left inferior pulmonary vein

### Procedural data

**Fig. 2:**
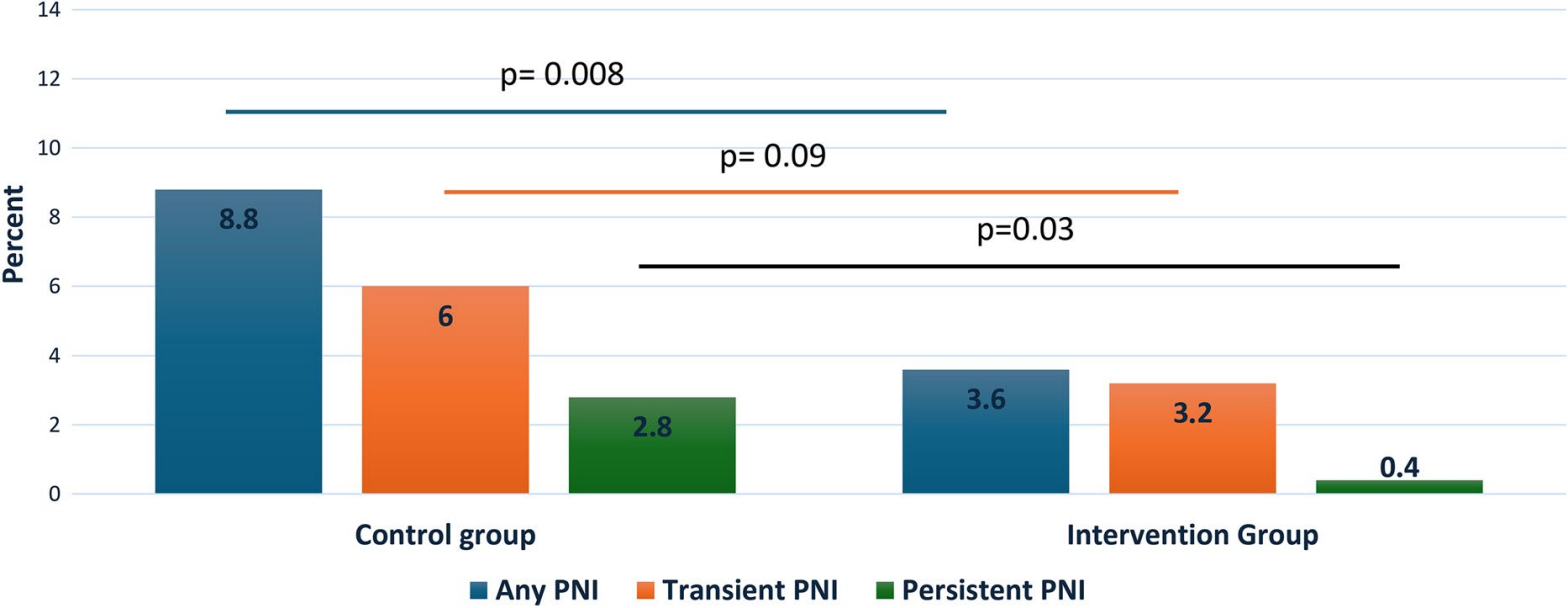
Differences in the incidence of phrenic nerve injury between two groups. *PNI* phrenic nerve injury

During the application of cryoenergy to RSPV, 66 out of 900 patients (7.3%) experienced PNI. Of those affected, 47 (5.2%) individuals recovered during the procedure, while the remaining 19 (2.1%) had persistent PN paresis at discharge. All of the PNIs occurred during the cryo therapy of the RSPV. We observed a statistically significant difference in terms of PNI between the two groups. The incidence of any PNI was lower in the intervention group (3.6% vs. 8.8%, *p* = 0.008) as well as persistent PNI (0.4% vs. 2.8%, *p* = 0.03) (Fig. 2). The incidence of transient PNI also tended to be lower in the intervention group (3.2% vs. 6.%, *p* = 0.09), The logistic regression analysis revealed that the utilization of the intervention was significantly associated with a reduced risk of any PNI (odds ratio (OR), 0.39; 95% CI [0.19–0.79]; *p* = 0.006). This association remained significant even after adjusting for age, gender, and type of AF (paroxysmal vs persistent) (OR, 0.36; 95% CI [0.18–0.74]; *p* = 0.007), please refer to Table 3. The PN threshold in the control group was analyzed and found to be 2.7 ± 2.6 V/2.0 ms. The average output for PN stimulation was 5.6 ± 5.2 V/2.0 ms, which is almost half of the regular output (10 V/2.0 ms).

**Table 3:** The logistic regression analysis

	Univariate OR (95% CI)	Univariate *p*-value	Multivariate* OR (95% CI)	Multivariate* p-value
Any PNI	0.39 [0.19–0.79]	0.01	0.36 [0.18–0.77]	0.007
Persistent PNI	0.14 [0.02–0.98]	0.048	0.14 [0.02–0.99]	0.05

*After adjustment for age, gender, type of AF (paroxysmal vs persistent)

*PNI* phrenic nerve injury

## Discussion

The CB technique for PVI has become the standard for first-line interventional treatment of AF due to its simplicity and safety. It is widely used in electrophysiology laboratories [8–10]. However, a common complication of this procedure is PNI. Although recent studies have described techniques to reduce PNI, it remains the most frequent complication [3–5]. We combined several approaches, added our own techniques, and systematized them in a stepwise approach.

### The steps to avoid PNI

The steps to avoid PNI are mostly based on recent publications and our own experience with techniques for reducing PNI. We have also added a new aspect: stimulating the PN with less output, closer to the threshold, instead of the standard 10 V output.

- **Step 1—Reduced output for PN stimulation: **The stimulation threshold for the phrenic nerve was detected in all patients in the interventional group. Instead of constantly pacing with a 10 V amplitude, we stimulated the phrenic nerve close to the threshold with double output for safety. The average output for phrenic nerve stimulation in the intervention group was almost half that of the control group. This allows for greater sensitivity in early recognition of PNI (see Fig. 1E).
- **Step 2—Pre-freezing for RSPV:** Saitoh et al. investigated the significance of the fluoroscopic position of the CB in the right superior PV as a predictor of phrenic nerve injury. They defined three positions for the lower half of the balloon: (A) completely inside the cardiac shadow, (B1) 1/3 outside the cardiac shadow, and (B2) ≥ 1/3 outside the cardiac shadow [11]. They demonstrated that the B2 position was the strongest independent predictor of PNI at RSPV, while the A position was associated with minimal risk. This can be explained by the anatomical relationship between the RSPV and PN. The balloon in the RSPV is more distant to the PN with the pre-freezing approach, which has been referred to as the “advance approach” for the right superior pulmonary vein. For this purpose, we first initiated cryotherapy in close proximity to the RSPV ostia. Then, we inserted the balloon towards the ostia under simultaneous angiography until the occlusion was verified. This technique was recently published by Tokuda et al. They used it to increase the surface of damaged/isolated tissue and advocated for its potential to yield better outcomes. The study did not investigate the influence on the PNI. However, it did mention a tendency for less incidence of CMAP reduction in the pre-freezing group. The authors suggest that pre-freezing leads to more antral adhesion of the balloon, thus avoiding close contact with the PN [12].
- **Step 3—Pull away for RSPV and RIPV:** Okishige et al. reported the importance of the pullback maneuver in reducing PNI. In their study, colleagues pulled back the CB once the reduction of the CMAP potential was registered at more than 30%, instead of stopping the application. The study clearly demonstrated that after this maneuver, the CMAP amplitude recovered in 85.9% of all cases, and the application was conducted uninterrupted. This is due to the anatomical relationship between the RSPV and PN. Once pulled back, the balloon stays at a safe distance from the PN [13]. We modified this approach as follows: after achieving − 40 °C and/or isolating the PV, a “pull-away” maneuver (similar to the pull-back maneuver, but holding in the pulled position until the end of the procedure) was performed for both septal PVs.

For the angiography of the pulmonary veins in the interventional group, we used our recently proposed safe, quick, and simple method. This method provides a sufficient overview of the entire left atrium, with a clear demonstration of the antral segments of the PVs and is performed under right ventricular rapid pacing using the SL1 sheath [6]. To avoid vagal response, we always started the applications with the right pulmonary veins and then the left pulmonary veins in the interventional group. Miyazaki et al. demonstrated significantly fewer marked vagal responses occurred when the left superior pulmonary vein (LSPV) was targeted after the right PVs [14]. This effect is due to the specific anatomy of the post-ganglionic parasympathetic innervation which is mainly located among the right pulmonary veins and the inter-atrial septum. Targeting this area leads to achieving vagal denervation and is also used for preventing inappropriate functional bradycardia and syncope recurrence in patients affected by neurally mediated syncope [15–17]. The impact of the angiography technique or targeting sequence of the pulmonary veins on reducing the PNI is uncertain.

Although the focus of the study was PNI, we observed significant differences in procedural time between the two groups. The interventional group had a shorter procedure time due to several factors. Firstly, operators’ experience has continuously increased. Secondly, in the interventional group, a quick PV-angiography technique was used, which resulted in a lower amount of contrast too. Thirdly, the intervention group used the TTI plus 120 s algorithm. Lastly, the PVI was standardized with a right-to-left sequence (“complex part first” approach) in the intervention group as described above.

All of these changes in the technique were made based on the historical development of the procedure and increased experience. This is the reason why patients who were ablated 7 years ago had a slightly different periprocedural course. Additionally, the ICE-guided procedures in the control group resulted in lower fluoroscopy time and radiation dose, which is also an important consideration for future modifications of the procedure. The data from the literature indicate that the PNI occurs, on average, at 120 s [3, 5, 11]. Therefore, the difference in application times between the two groups (RSPV, 191 s vs 240 s; RIPV, 200 s vs 270 s) may not be a significant factor in comparing the PNI incidents.

All presented novel steps do not require extra time or special skills. They are easy to perform and effective in improving procedure safety and minimizing PNI. Despite the development of new technologies and energy applications in recent years, addressing the Achilles heel may help cryoballoon therapy remain relevant for decades to come.

### Study limitations

This is a non-randomized study conducted by only two centers. The number of patients is quite high, but there are some differences in the technical approaches between the two groups, which, however, should not affect the main focus of the study.

## Conclusion

This retrospective study provides insights into the effectiveness of our novel technique in significantly reduction of the incidence of PNI, which is the most frequent major complication of CB-PVI. This approach may reduce the incidence of the persistent PNI to unprecedented levels.
